# Impact of tongue fat volume on obstructive sleep apnea in non-obese patients

**DOI:** 10.1038/s41598-025-08747-z

**Published:** 2025-09-30

**Authors:** Michael I. Orestes, Kenneth R. Feehs, Gregory S. Hill, Robert Shih, Jacob F. Collen, Emily A. Montgomery, Katelyn M. Waring, Nora L. Watson, Richard W. Thomas, Lilit Garibyan

**Affiliations:** 1 Department of Otolaryngology-Head & Neck Surgery, Walter Reed National Military Medical Center, Bethesda, MD USA; 2 Department of Surgery, Uniformed Services University of the Health Sciences, Bethesda, MD USA; 3 Department of Otolaryngology, Wake Forest University School of Medicine, Winston-Salem, NC USA; 4 Department of Radiology, Uniformed Services University of the Health Sciences, Bethesda, MD USA; 5 Department of Sleep Medicine, Walter Reed National Military Medical Center, Bethesda, MD USA; 6 School of Medicine, Uniformed Services University of the Health Sciences, Bethesda, MD USA; 7 Department of Dermatology, Harvard Medical School, Boston, MA USA

**Keywords:** Sleep apnea, OSA, Tongue fat, MRI, Obesity, Magnetic resonance imaging, Obesity, Body mass index

## Abstract

Evidence suggests excess head and neck adipose tissue contributes to OSA, particularly in obese patients. Surgical treatments are often ineffective in this subset of the population. We sought to investigate the role of tongue fat in the normal and overweight populations. In this prospective cohort study, patients underwent overnight polysomnography and MRI using a Dixon sequence. Volumetric reconstruction evaluated the size and distribution of tongue fat deposits in subjects with and without sleep apnea. The study included 86 patients; mean age of 42.2 (SD 11.2) years, 16% female. Average BMI 27.5 kg/m^2^ (SD 2.9), with 18.6% (*n* = 16) normal BMI, 61.6% (*n* = 53) overweight, 19.8% (*n* = 17) obese. Logistic regression lines showed positive associations for BMI and age with AHI. No significant correlation was found between tongue fat volume or fraction and increased AHI nor presence of OSA. Although tongue volume and fat fraction were higher in patients with AHI ≥ 5 events per hour, the difference was not statistically significant. This study suggests that tongue fat does not play a significant role in the pathophysiology of OSA in the non-obese (BMI < 30) patient population. Therefore, selective treatments targeting tongue fat should focus on obese and morbidly obese patients.

**Supplementary Information:**

The online version contains supplementary material available at 10.1038/s41598-025-08747-z.

## Background

Obstructive sleep apnea is a chronic medical condition that can lead to excessive daytime sleepiness, cognitive impairment, and an increased risk of motor vehicle accidents^1,2^. The underlying causes of OSA are multifaceted, including anatomic obstruction, inadequate dilation of dilator muscles during sleep, and an oversensitive ventilatory control system^3^. There is a growing body of evidence suggesting that excess adipose tissue within the head and neck contributes to OSA, particularly in obese patients^4–6^. Recent studies have shown that weight loss by lifestyle modification or bariatric surgery in obese patients with OSA patients leads to a reduction in tongue fat volume as observed on MRI, which is associated with a decrease in apnea-hypopnea index, though these studies defined OSA as AHI > 10 events per hour on polysomnogram^4,7^. While the mechanism through which AHI is reduced remains unclear, it is theorized that reduction of tongue fat may increase the retroglossal airway space or improve the protrusion ability of the tongue. Previous research has suggested that high fat content in muscles may impair their effectiveness^8^. However, a recent study examining the relationship between tongue fat and tongue protrusion found that patients with more severe OSA had larger tongue volumes and greater inspiratory tongue movement, indicating that tongue protrusion ability is not affected by increased tongue fat^9^. Furthermore, one study reported higher levels of tongue fat in obese individuals with OSA compared to obese individuals without OSA, suggesting that the phenotype of OSA in obese patients may differ from non-obese patients^10^. These findings may provide further insight as to why OSA in obese patients is notoriously difficult to treat^11^. Current mainstream hypopharyngeal surgeries include tongue radiofrequency ablation, midline glossectomy, genioglossus advancement, lingual tonsillectomy and hypoid suspension. Hypopharyngeal procedures constitute nearly 35% of sleep apnea inpatient procedures in the US^12^. Procedures specifically designed to reduce tongue volume, such as tongue radiofrequency, midline glossectomy, and lingual tonsillectomy comprised the majority of these, with tongue base radiofrequency being the most common^12^. However, this procedure is associated with a 93% rate of significant postoperative pain^13^. In this study, we aimed to further investigate the role of tongue fat in OSA with our main focus on non-obese patients and hypothesized that there would be a significant difference in tongue fat volume between obese and non-obese patients with OSA.

## Objective

To assess for an association between tongue fat and AHI in non-obese and mildly obese patients.

## Study design and methods

This study was conducted in accordance with the ethical guidelines provided by the Walter Reed National Military Medical Center (WRNMMC) Institutional Review Board (IRB). The study protocol was approved by the IRB (#2018-BOT). Written informed consent was obtained from all participants prior to their inclusion in the study.

In this prospective cohort study, patients were recruited immediately after undergoing an overnight type 1, fully attended, seven channel in lab polysomnography at an American Academy of Sleep Medicine (AASM) accredited lab at a single institution over a 24-month period, in compliance with the approved institutional IRB protocol. The investigators were blinded to whether the patients had OSA.

Recruited subjects meeting inclusion criteria then underwent MRI of the face utilizing a two-point Dixon sequence to separate water from fat signal. All MRIs were performed on a 3.0-T scanner within one week of PSG, prior to final interpretation of the patients’ PSG. Image analysis with Amira Software 2020.3.1 (Thermo Fisher Scientific, Waltham, MA) was performed by a neuroradiologist with 15 years of experience in head and neck imaging, who was blinded to patient BMI and PSG results.

Eligible participants included adults ≥ 18 year of age eligible for care at military treatment facility undergoing a PSG for evaluation of sleep apnea who could obtain an MRI. We excluded those with a history of head and/or neck radiation, history of tongue surgery, history of oropharyngeal cancer, current pregnancy, or those with a relative or absolute contraindication to undergoing MRI. None of the enrolled participants had a history of stroke resulting in tongue paralysis. One subject was excluded due to dental braces and severe metallic susceptibility artifact obscuring tongue visualization on face MRI. The study size was determined to be adequate via comparative results by reproducing known, significant associations at the end of the study period.

**Fig. 1:**
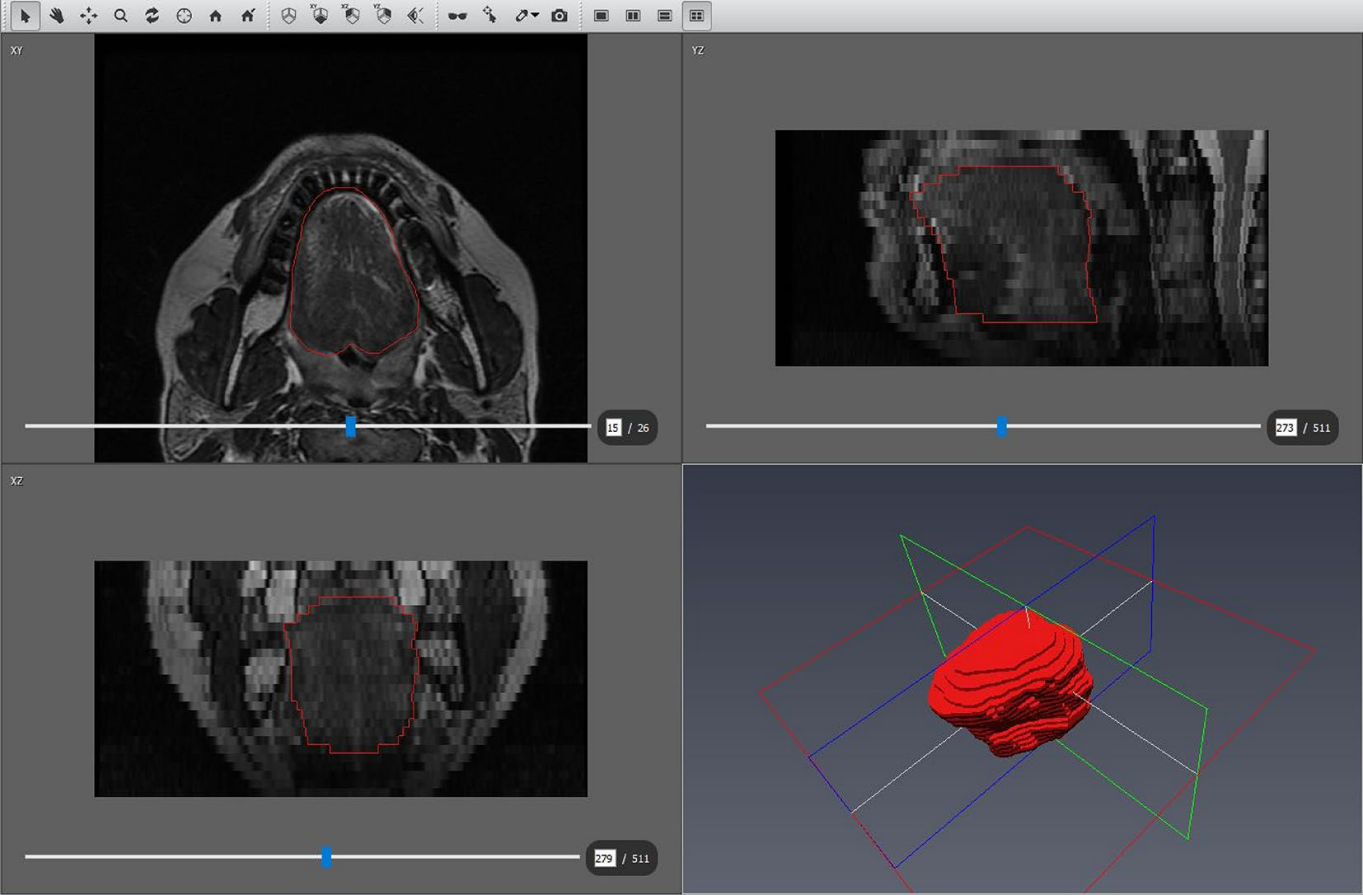
Tongue fat tissue volumetric analysis. Example screenshot of Amira image analysis software to segment the tongue and to measure total tongue volume (mL) on the in-phase T2-weighted images with multiplanar reformats. Mean signal in the tongue volume on the fat-only images (not shown) was divided by mean signal in parapharyngeal adipose tissue to yield an estimate of tongue fat fraction (%), which was then multiplied by total tongue volume to yield an estimate of tongue fat volume (mL) for each subject. Values are reported in Tables 1 and 2.

Demographics including age and sex were collected by chart review. Height and weight were measured at the time of PSG, and BMI was calculated from these measurements. BMI was classified according to Adult BMI CDC guidelines, with obesity subdivided into Class 1 (BMI of 30 to < 35 kg/m^2^), Class 2 (BMI 35 to < 40 kg/m^2^) and Class 3 (BMI of 40 or higher kg/m^2^)^14^. Outcomes including apnea-hypopnea index (AHI) and OSA were defined in accordance with the AASM 2017 Adult OSA Clinical Practice Guideline. Using a 3.0 Tesla scanner, high-resolution 1.5T MRI of the head and neck from top of the skull to the clavicles without contrast was performed with subject’s head in neutral position to image the oral cavity and oropharynx. Subjects were provided standard instructions for MRI of the head and neck. Soft tissue volumes were determined from T1 axial image and axial T2-weighted Dixon MRI sequences were utilized to separate water from fat signal (in-plane resolution 0.35 × 0.35 mm and slice thickness 3 mm) to allow volumetric assessment of adipose tissue within the tongue. For each MRI, our neuroradiologist used Amira image analysis software to segment the tongue and to measure total tongue volume (mL) on the in-phase images with multiplanar reformats. Mean signal in the tongue volume on the fat-only images was divided by mean signal in parapharyngeal adipose tissue to yield an estimate of tongue fat fraction (%), which was then multiplied by total tongue volume to yield an estimate of tongue fat volume (mL) for each subject (Fig. 1).

Multiple logistic regression testing including Fisher Exact and Wilcox rank sum tests were performed to assess for associations among demographics, BMI, AHI, tongue volume, tongue fat volume, and tongue fat fraction. Volumetric analyses of patients with OSA were compared to their matched controls without. Results are reported in accordance with STROBE guidelines.

## Results

A total of 86 patients met inclusion criteria, with all patients completing both a PSG and MRI. The mean age of this cohort was 42.2 (SD, 11.2) years, 81.4% male (*n* = 70) and 18.6% female (*n* = 16). The average BMI was 27.5 kg/m^2^ (SD, 2.89), with 18.6% (*n* = 16) normal BMI, 61.6% (*n* = 53) overweight, 19.8% (*n* = 17) obese (Table 1). All patients had BMI less than 35 kg/m^2^.

**Table 1:** Characteristics by OSA severity.

	Normal	Mild	Moderate	Severe	Overall
	(*n* = 36)	(*n* = 25)	(*n* = 14)	(*n* = 9)	(*n* = 86)
Age					
Mean (SD)	38.2 (10.9)	44.7 (10.1)	41.9 (10.9)	52.4 (9.03)	42.2 (11.2)
Median [Min, Max]	38.0 [20.0, 61.0]	42.0 [30.0, 71.0]	42.0 [25.0, 66.0]	55.0 [31.0, 60.0]	42.0 [20.0, 71.0]
Gender					
Female	8 (22.2%)	3 (12.0%)	3 (21.4%)	1 (11.1%)	16 (18.6%)
Male	28 (77.8%)	22 (88.0%)	11 (78.6%)	8 (88.9%)	70 (81.4%)
BMI (kg/m^2^)					
Mean (SD)	26.5 (2.64)	28.0 (2.90)	28.1 (3.27)	29.5 (1.67)	27.5 (2.89)
Median [Min, Max]	26.5 [19.6, 32.8]	28.2 [22.5, 34.0]	28.4 [22.1, 33.6]	29.2 [27.3, 32.1]	27.2 [19.6, 34.0]
BMI category					
Normal	9 (25.0%)	4 (16.0%)	2 (14.3%)	0 (0%)	16 (18.6%)
Overweight	23 (63.9%)	15 (60.0%)	8 (57.1%)	6 (66.7%)	53 (61.6%)
Obese	4 (11.1%)	6 (24.0%)	4 (28.6%)	3 (33.3%)	17 (19.8%)
Height (in)					
Mean (SD)	69.7 (3.28)	70.3 (3.05)	70.3 (3.75)	68.8 (2.54)	69.9 (3.20)
Median [Min, Max]	70.0 [63.0, 76.0]	70.0 [65.0, 76.5]	70.0 [62.0, 76.0]	69.0 [65.0, 73.0]	70.0 [62.0, 76.5]
Weight (lbs)					
Mean (SD)	186 (22.6)	198 (30.3)	199 (26.7)	198 (11.9)	193 (25.6)
Median [Min, Max]	186 [129, 231]	203 [135, 265]	209 [150, 235]	199 [186, 220]	191 [129, 265]
Tongue volume					
Mean (SD)	94,200 (15,200)	98,300 (17,600)	103,000 (14,700)	97,300 (8,300)	96,800 (15,600)
Median [Min, Max]	95,400 [56,700, 121,000]	94,700 [61,900, 140,000]	107,000 [75,700, 124,000]	94,700 [88,000, 111,000]	95,700 [56,700, 140,000]
Mean signal					
Mean (SD)	637 (404)	623 (452)	732 (418)	589 (426)	644 (419)
Median [Min, Max]	742 [161, 1330]	303 [121, 1490]	924 [139, 1250]	312 [213, 1140]	734 [121, 1490]
Fat signal					
Mean (SD)	2,100 (1,200)	1,980 (1,230)	2,340 (1,150)	1,930 (1,210)	2,080 (1,190)
Median [Min, Max]	2,700 [464, 3,710]	1,330 [505, 4,070]	2,880 [741, 3,690]	1,110 [737, 3,510]	2,610 [464, 4,070]
Fat fraction					
Mean (SD)	0.300 (0.0672)	0.303 (0.0869)	0.295 (0.0724)	0.291 (0.0677)	0.301 (0.0733)
Median [Min, Max]	0.302 [0.152, 0.493]	0.276 [0.159, 0.491]	0.299 [0.147, 0.384]	0.286 [0.195, 0.423]	0.302 [0.147, 0.493]
Fat Volume					
Mean (SD)	28,500 (9,080)	29,800 (9,870)	30,200 (7,490)	28,300 (6,530)	29,100 (8,640)
Median [Min, Max]	27,600 [13,000, 47,900]	29,100 [14,600, 47,200]	32,600 [16,500, 40,600]	28,000 [19,800, 40,100]	28,200 [13,000, 47,900]

Demographics of study population including age and gender, measured values of BMI, height, and weight, and volumetric analyses including tongue volume, fat signal, fat fraction, and fat volume are stratified by OSA severity. Values generated by volumetric analyses are unitless.

**Table 2:** Characteristics by presence or absence of OSA.

	AHI < 5 events per hour	AHI ≥ 5 events per hour	Overall
	(*n* = 36)	(*n* = 48)	(*n* = 86)
Age			
Mean (SD)	38.2 (10.9)	45.4 (10.6)	42.2 (11.2)
Median [Min, Max]	38.0 [20.0, 61.0]	43.0 [25.0, 71.0]	42.0 [20.0, 71.0]
Gender			
Female	8 (22.2%)	7 (14.6%)	16 (18.6%)
Male	28 (77.8%)	41 (85.4%)	70 (81.4%)
BMI (kg/m^2^)			
Mean (SD)	26.5 (2.64)	28.3 (2.84)	27.5 (2.89)
Median [Min, Max]	26.5 [19.6, 32.8]	28.4 [22.1, 34.0]	27.2 [19.6, 34.0]
BMI category			
Normal	9 (25.0%)	6 (12.5%)	16 (18.6%)
Overweight or obese	27 (75.0%)	42 (87.5%)	70 (81.4%)
Height (in)			
Mean (SD)	69.7 (3.28)	70.0 (3.18)	69.9 (3.20)
Median [Min, Max]	70.0 [63.0, 76.0]	70.0 [62.0, 76.5]	70.0 [62.0, 76.5]
Weight (lbs)			
Mean (SD)	186 (22.6)	198 (26.3)	193 (25.6)
Median [Min, Max]	186 [129, 231]	203 [135, 265]	191 [129, 265]
Tongue volume			
Mean (SD)	94,200 (15,200)	99,600 (15,300)	96,800 (15,600)
Median [Min, Max]	95,400 [56,700, 121,000]	96,600 [61,900, 140,000]	95,700 [56,700, 140,000]
Mean signal			
Mean (SD)	637 (404)	648 (432)	644 (419)
Median [Min, Max]	742 [161, 1,330]	720 [121, 1,490]	734 [121, 1,490]
Fat signal			
Mean (SD)	2,100 (1,200)	2,080 (1,190)	2,080 (1,190)
Median [Min, Max]	2,700 [464, 3,710]	2,450 [505, 4,070]	2,610 [464, 4,070]
Fat fraction			
Mean (SD)	0.300 (0.0672)	0.299 (0.0782)	0.301 (0.0733)
Median [Min, Max]	0.302 [0.152, 0.493]	0.293 [0.147, 0.491]	0.302 [0.147, 0.493]
Fat volume			
Mean (SD)	28,500 (9,080)	29,600 (8,540)	29,100 (8,640)
Median [Min, Max]	27,600 [13,000, 47,900]	28,700 [14,600, 47,200]	28,200 [13,000, 47,900]

Demographics of study population including age and gender, measured values of BMI, height, and weight, and volumetric analyses including tongue volume, fat signal, fat fraction, and fat volume are stratified by the presence or absence of sleep apnea. Values generated by volumetric analyses are unitless.

Measured variables from PSG data included AHI, from which classification of OSA was determined. Measured variables from Amira image analysis of the face MRIs included tongue volume (mL), mean tongue signal (unitless), mean adipose signal (unitless), tongue fat fraction (%), and tongue fat volume (mL).

The median AHI was 7.4 events per hour (IQR, 12.9), with 41.9% (*n* = 36) without OSA, 29.1% (25) mild OSA, 16.3% (14) moderate OSA, and 10.5% (9) severe OSA.

Fisher Exact testing was performed to assess for significant associations of gender and BMI category with OSA, with no significant associations found.

**Fig. 2:**
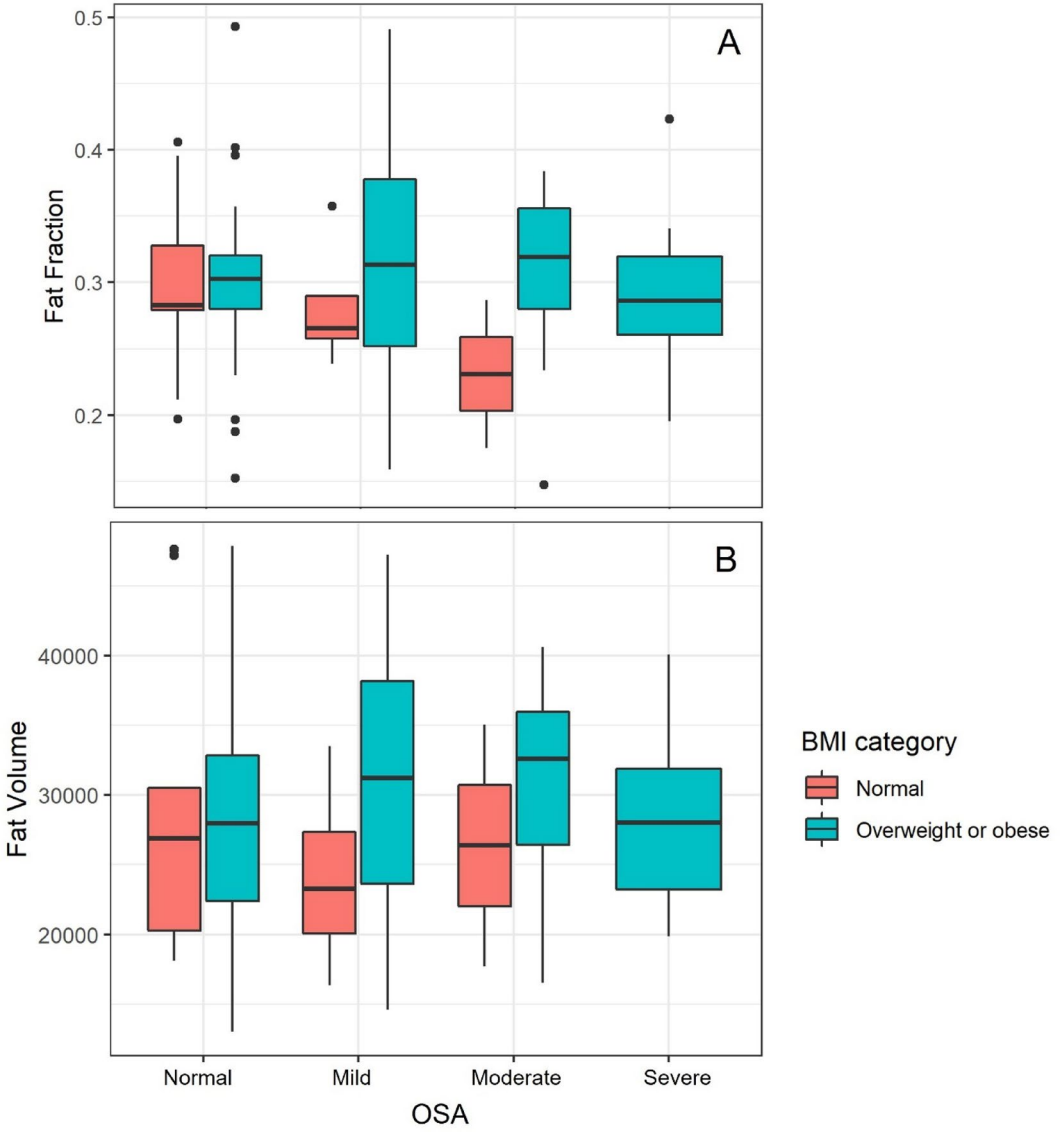
Tongue fat contribution to AHI in normal weight vs. overweight and obese patients. Box and whiskers plots of fat fraction (**A**) and fat volume (**B**) vs. AHI, when stratified into groups of patients of normal weight and overweight + obese patients. No statistically significant difference in tongue fat fraction or fat volume was found between these groups (*P* > 0.05).

**Fig. 3:**
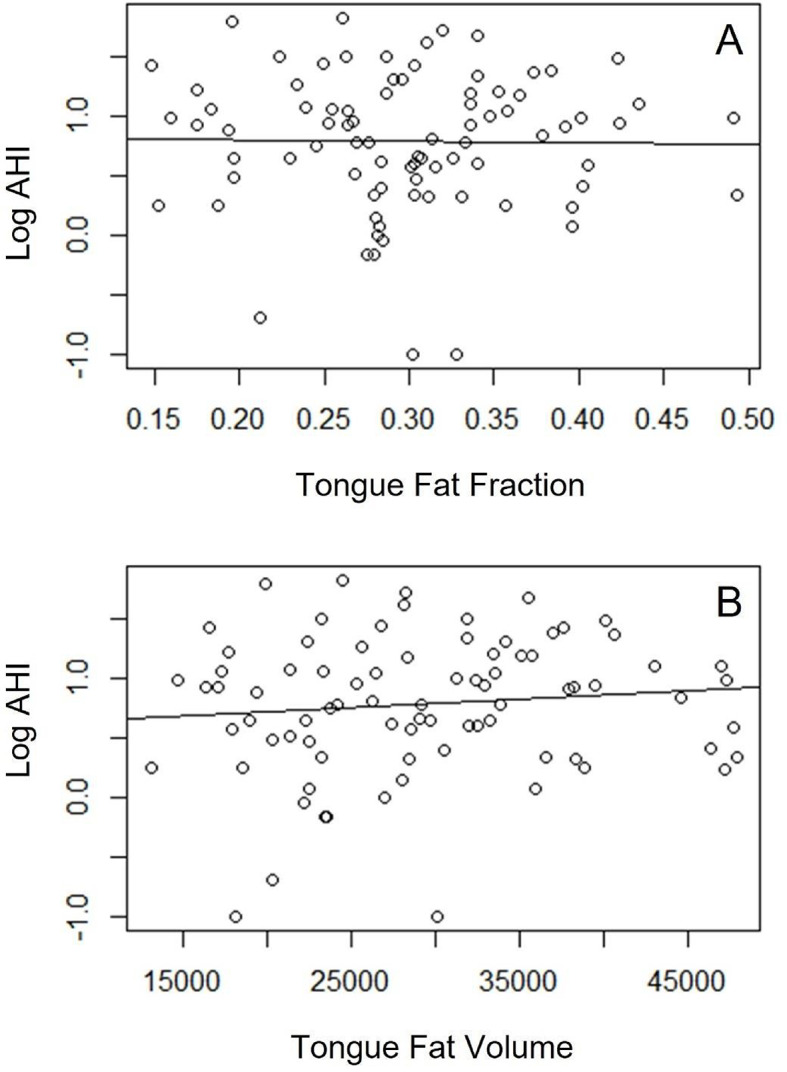
Logistic regression analyses of tongue fat versus AHI. Logistic regression analyses were performed given non-parametric distribution of AHI values in this study population. Regression lines for fat fraction (**A**) and fat volume (**B**) compared with AHI are shown. No statistically significant correlation was noted (*P* > 0.05).

**Fig. 4:**
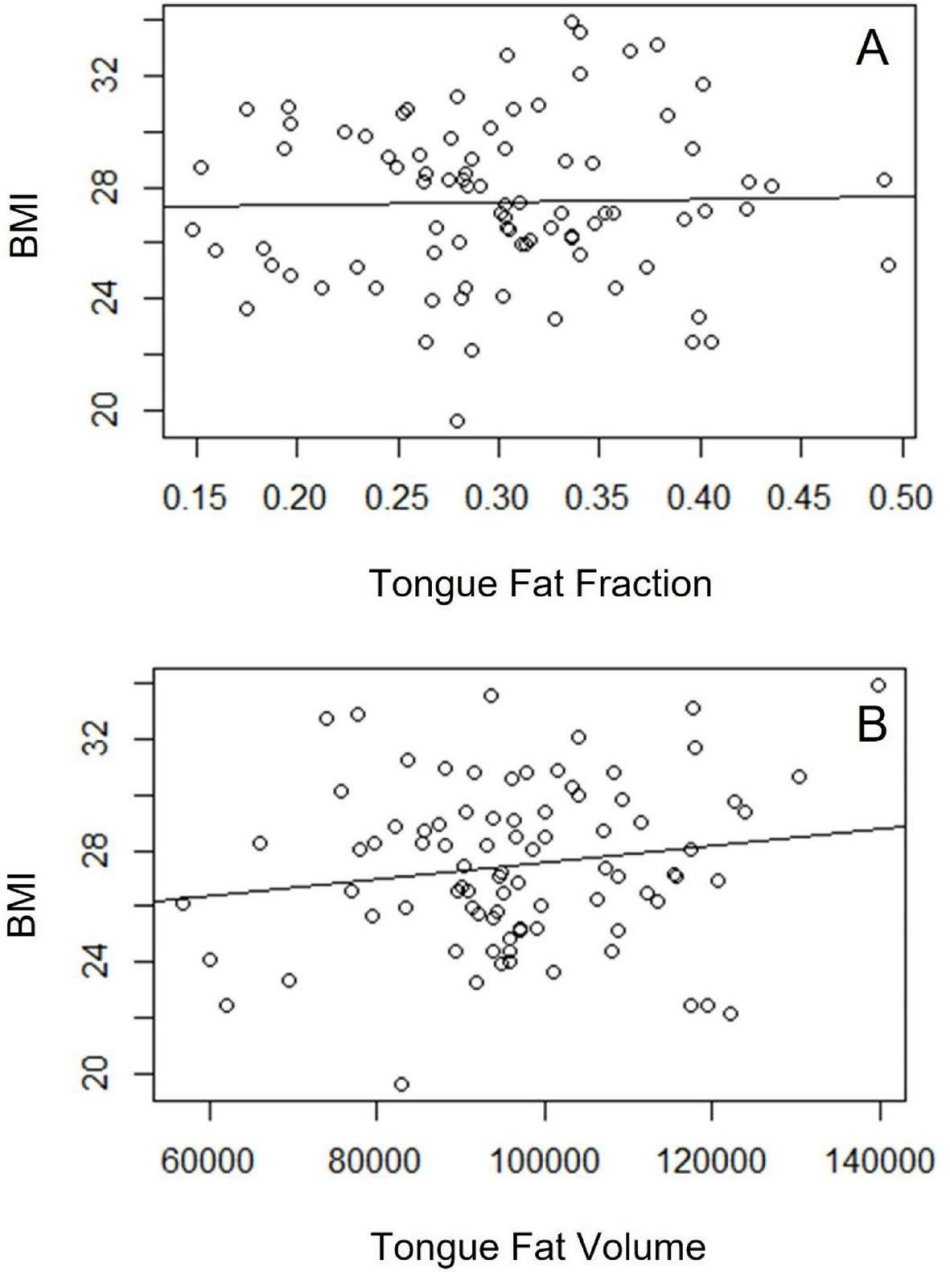
Regression analyses of tongue fat and tongue volume versus BMI. Regression lines for tongue fat fraction (**A**) and tongue volume (**B**) compared with BMI are shown. No statistically significant correlation was noted (*P* > 0.05).

Wilcox rank sum test was performed to assess for significant associations of demographic and measured variables with OSA. Whereas tongue volume, mean tongue signal, tongue fat fraction, and tongue fat volume were not associated with OSA (*p* > 0.05) (Fig. 2), demographic variables of age and BMI value were associated with OSA (*p* < 0.05, *p* < 0.005, respectively). To further explore these associations, we plotted logistic regression lines comparing age and BMI to normalized AHI values. We found positive, significant correlations for both age (r^2^ 0.12, *p* < 0.005, SE 0.02) and BMI (r^2^ 0.14, *p* < 0.001, SE 0.005). Overall, patients with OSA (AHI ≥ 5 events per hour, *n* = 48) demonstrated higher average age (45.4 years) and BMI (28.3) compared those without OSA (AHI < 5 events per hour, *n* = 36) who had an average age (38.2 years) and BMI (26.5) in those with AHI < 5. Regression lines were plotted to assess for any correlation of our volumetric measurements with BMI or AHI (Figs. 3 and 4). No significant correlations were found between BMI and tongue volume nor tongue fat fraction. Likewise, no significant correlations were found between AHI and tongue fat fraction nor tongue fat volume. Finally, we used a linear regression model to assess for correlations between tongue fat fraction and normalized AHI values while adjusting for both BMI and age, and did not find a significant correlation. A similar model assessing correlations between tongue fat fraction and the presence or absence of OSA showed a higher tongue fat fraction in patients with OSA, but this was not statistically significant.

## Discussion

Our study suggests that excess tongue fat does not appear to play a significant role in the pathophysiology of OSA in the non-obese or overweight (BMI < 30 kg/m^2^) patient population. While we also found that tongue fat volume was not associated with mild obesity (BMI 30-34.9), the mildly obese population in this study was small (*n* = 17). There were no significant correlations between tongue fat volume and AHI nor BMI. We did find significant positive correlations of OSA with age and BMI in our study population. This correlation has been previously described in the literature, but mainly in very obese patients, which is different from the population we studied here.

In contrast to previous studies of tongue fat and its relationship to obstructive sleep apnea, our study is unique in that it assesses this relationship in a lower BMI population. No patients in our cohort had a BMI ≥ 35. In fact, only about 20% of our study population was obese compared to roughly 40% of the US adult population. This likely reflects the well described phenomenon of a lower prevalence of obesity in military communities compared to the general population^15^. When analyzed in context with existing literature on tongue fat in patients with OSA, our data suggests that tongue fat is not a significant contributor to the development of OSA in non-obese or overweight patient populations, as these patients did not have a significant difference in tongue fat volume or tongue fat fraction compared to matched controls without OSA. A recent study examining tongue fat volume with both MRI and ultrasound similarly found no difference in overall tongue fat volume or percentage between mildly obese patients (average BMI 31 kg/m^2^) and normal BMI controls, noting only a difference in tongue midsagittal area fat volume^16^. Given that prior studies have demonstrated a correlation between tongue fat volume and OSA in obese patients—both of which improved with weight loss—it is possible that tongue fat plays a significant role of OSA in the obese population, and may represent a distinct phenotype^4,17^. One of these studies using similar MRI volumetric analyses demonstrated that weight loss in obese patients with OSA resulted in significant reductions in parapharyngeal, retropharyngeal, and base of tongue fat, and these reductions were strongly associated with AHI reduction, with base of tongue fat volume reduction showing the strongest association^4^. However, this study defined OSA as AHI > 10 events per hour, whereas in this study we maintain the AASM definition of AHI ≥ 5 events per hour. Our findings suggest that such results would not be expected in a non-obese population, though our study does not suggest what other factors may contribute to a non-obese phenotype of OSA. Future studies should explore other structural and/or functional contributors to OSA in a non-obese population.

In the obese population, the role of tongue fat as a contributor to OSA is well described. This may help to explain why obese and morbidly obese patients are difficult to treat, as the most commonly used current therapies do not target tongue fat^18,19^. While weight loss appears to be beneficial, weight loss is a difficult goal to achieve, with an estimated median success rate of 15%.^20^ A recent small randomized control trial demonstrated a significant reduction in tongue volume and associated reductions in body weight, BMI, and waist circumference in obese women after treatment with semaglutide (Ozempic) versus placebo^21^. Bariatric surgery has also been described to reduce body weight, improve the volume of the velopharyngeal airway, and decrease the volume of the tongue fat and pharyngeal fat^22^. Current surgical therapies for this population are associated with poor surgical outcomes^11^. The most common procedure used to address tongue volume is radiofrequency ablation, which was demonstrated in a systematic review to reduce ESS and RDI by 31% in the first year after surgery, though no differentiation was made by BMI^23^. A recent systematic review and meta-analysis of tongue base surgery complications demonstrated a mean complication rate of 12.79%, with a 4.4% rate for tongue base radiofrequency ablation and 42.42% rate for tongue base ablation^24^. These treatments often require repeated procedures to obtain a favorable outcome and are associated with high rates of postoperative pain^13,25^. Future therapies that selectively target tongue fat in a minimally invasive and practical way may be helpful in this population. A recent study evaluating the safety and feasibility of selective tongue fat reduction with ultrasound-guided base of tongue ice slurry injection demonstrated that the technology was feasible and well tolerated in a preclinical swine model, without histologic evidence of neurovascular damage nor airway compromise^26^. Further study into selective therapies for base of tongue fat reduction may prove to play a key role in OSA management for obese patients. Some limitations of this prospective cohort study include a somewhat small sample size, especially for mildly obese patients and lack of patients with BMI > 35. It is possible that a there is a small difference in tongue fat volume does indeed exist between mildly obese and non-obese patients with OSA, but that the power of our study was inadequate to reveal it given our sample size. However, we were able to demonstrate a statistically significant correlation between BMI and AHI along with age and AHI., a well described correlation in the literature^27^. With respect to the BMI of our population, we did not assess the impact of tongue fat in these moderate-severely obese patients. However, the goal of our study was to explore the role of tongue fat as a contributor to OSA in a lower BMI population.

## Conclusions

Tongue fat does not play a significant role in the pathophysiology of OSA in the non-obese (BMI < 30 kg/m^2^) patient populations. Future studies should explore the role of tongue fat in moderate and severely obese patients (BMI ≥ 35 kg/m^2^) with OSA, as well as other structural and/or functional contributors to OSA in the non-obese population.

## Electronic supplementary material

Below is the link to the electronic supplementary material.

Supplementary Material 1..

## Data Availability

The datasets generated during the current study are not publicly available to due HIPAA regulations but are available from the corresponding author on reasonable, HIPAA-compliant request.

## Abbreviations

OSA: Obstructive sleep apnea
WRNNMC: Walter Reed National Military Medical Center
BMI: Body mass index
AHI: Apnea-hypopnea index
MRI: Magnetic resonance imaging
PSG: Polysomnography
AASM: American Academy of Sleep Medicine
