# Supplementary material for: Impact of tongue fat volume on obstructive sleep apnea in non-obese patients
Source: Sci Rep. 2025 Sep 30;15:33924. doi: 10.1038/s41598-025-08747-z (PMC12484950; doi:10.1038/s41598-025-08747-z)
Supplement: Supplementary file 1 — Supplementary Material 1. [file 41598_2025_8747_MOESM1_ESM.docx]

S1. Logistic regression analyses of tongue fat versus AHI.


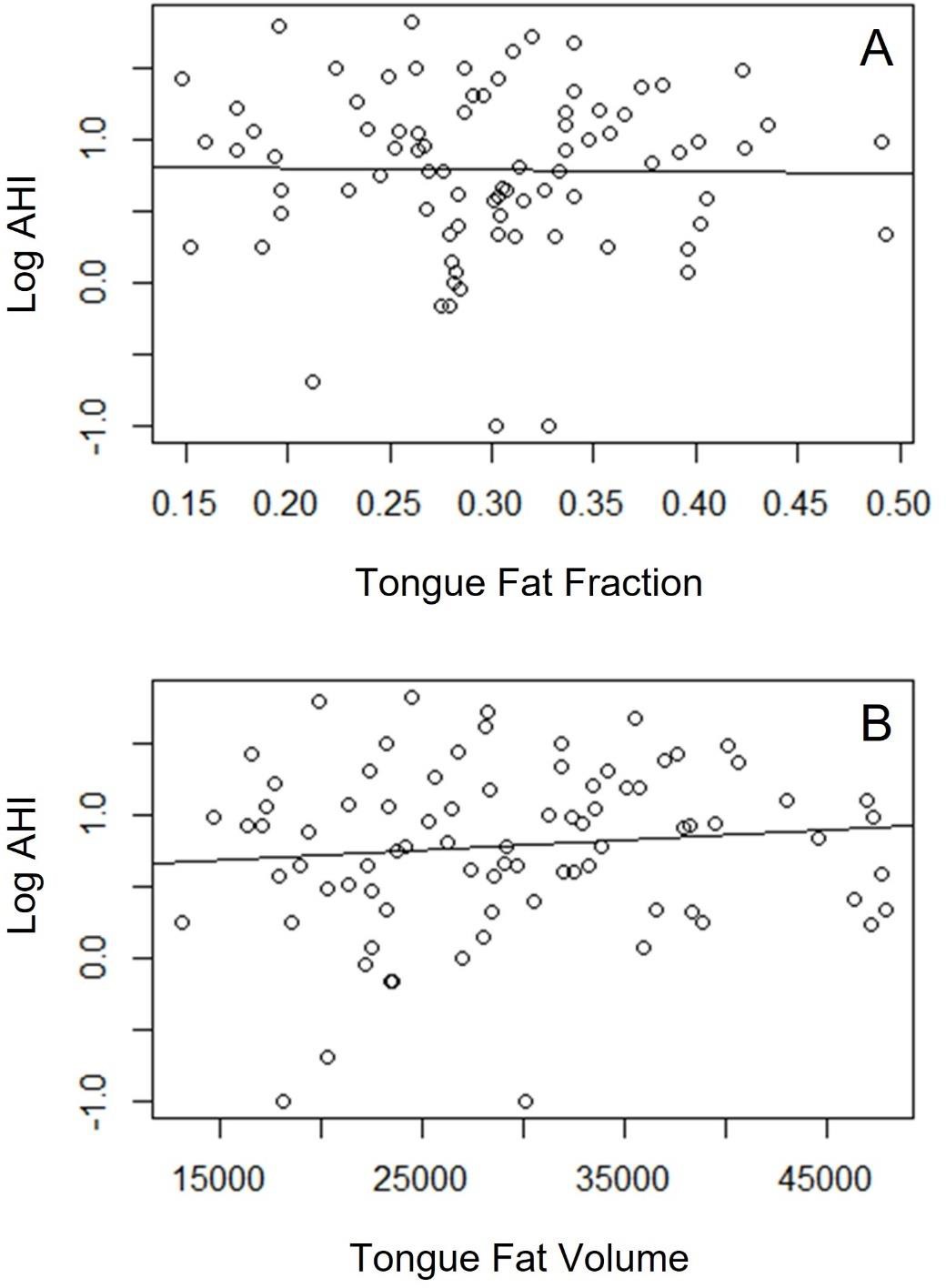


S2. Logistic regression analyses of tongue fat and tongue volume versus BMI.


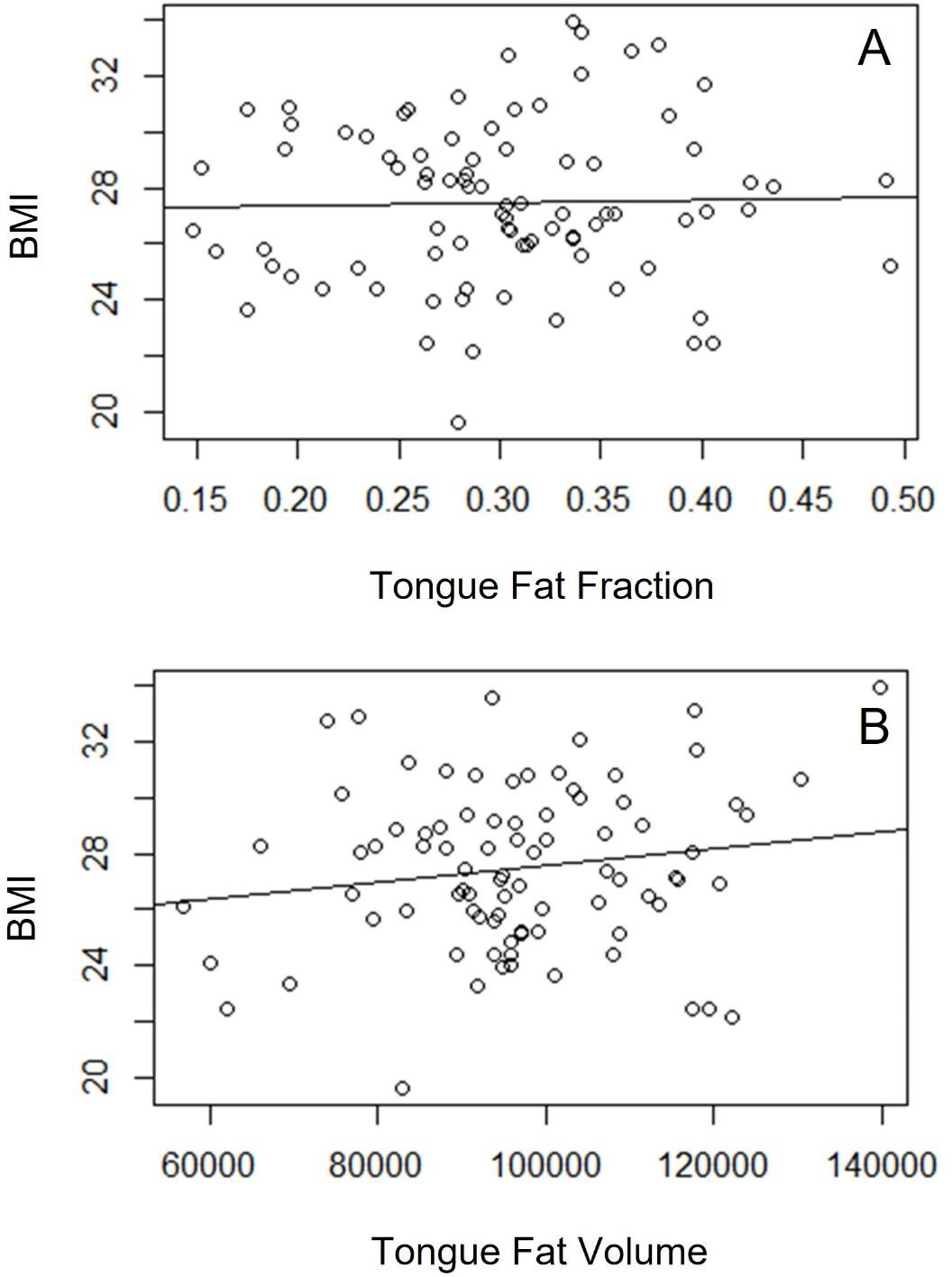


**Supplementary Figure Legend:**

**S1. Logistic regression analyses of tongue fat versus AHI.** Logistic regression analyses were performed given non-parametric distribution of AHI values in this study population. Regression lines for fat fraction (A) and fat volume (B) compared with AHI are shown. No statistically significant correlation was noted (P>0.05).

**S2. Regression analyses of tongue fat and tongue volume versus BMI.** Regression lines for tongue fat fraction (A) and tongue volume (B) compared with BMI are shown. No statistically significant correlation was noted (P>0.05).
